# Congenital cytomegalovirus in Sub-Saharan Africa—a narrative review with practice recommendations

**DOI:** 10.3389/fpubh.2024.1359663

**Published:** 2024-05-15

**Authors:** Helen Payne, Shaun Barnabas

**Affiliations:** ^1^Department of Paediatrics and Child Health, Faculty of Medicine and Health Sciences, University of Stellenbosch, Cape Town, South Africa; ^2^Section of Paediatric Infectious Disease, Imperial College London, London, United Kingdom

**Keywords:** congenital, cytomegalovirus, hearing loss, Africa, developmental impairment

## Abstract

Cytomegalovirus (CMV) is the most common cause of congenital infection internationally, occurring in 0.67% of births, and increasingly recognised as a major public health burden due to the potential for long-term neurodevelopmental and hearing impairment. This burden includes estimates of 10% of childhood cerebral palsy and up to 25% of childhood deafness. In Sub-Saharan Africa, where CMV-seroprevalence is almost ubiquitous, prevalence of congenital CMV (cCMV) is higher than the global average, and yet there is a dearth of research and initiatives to improve recognition, diagnosis and treatment. This narrative review outlines the epidemiology and clinical presentation of cCMV, discusses issues of case identification and treatment in Sub-Saharan Africa, and recommends a framework of strategies to address these challenges. Considering the significant burden of cCMV disease in this setting, it is undoubtably time we embark upon improving diagnosis and care for these infants.

## Epidemiology of cCMV

Cytomegalovirus (CMV) is a DNA herpesvirus that causes a typically mild or asymptomatic infection in an immunocompetent host and establishes lifelong persistent infection after primary infection (1). However, in immunocompromised hosts and the foetus, sequalae of CMV infection can be severe, life-long and even fatal. It was initially thought that the severity and frequency of cCMV disease was higher following maternal primary infection during pregnancy (2, 3). The high CMV seroprevalence found in women of childbearing potential in Sub-Saharan Africa (4) gave rise to a view that the impact of cCMV would be low (5). However, large Brazilian studies have since shown that maternal pre-conceptional immunity to CMV does not protect from cCMV, and that foetal infection can be due to either reactivation of latent infection or reinfection with new strains of CMV during pregnancy, known as non-primary infection (6). In fact, the prevalence of cCMV infection has been shown to increase as maternal CMV seroprevalence increases (6). Three-quarters of cCMV infections are now thought to be following non-primary infection with one-quarter due to primary infection (7). These findings are reinforced by a recent meta-analysis that estimated the rates of cCMV to be 3-times greater in low-and middle-income countries (LMICs), with a high CMV seroprevalence, than in high-income countries (HICs) where CMV seroprevalence is low (8). 85–95% of children in LMICs are seropositive for CMV by age 5–6 years (7), and so estimates for women of child-bearing age are close to 100% (9, 10).

**Figure 1:**
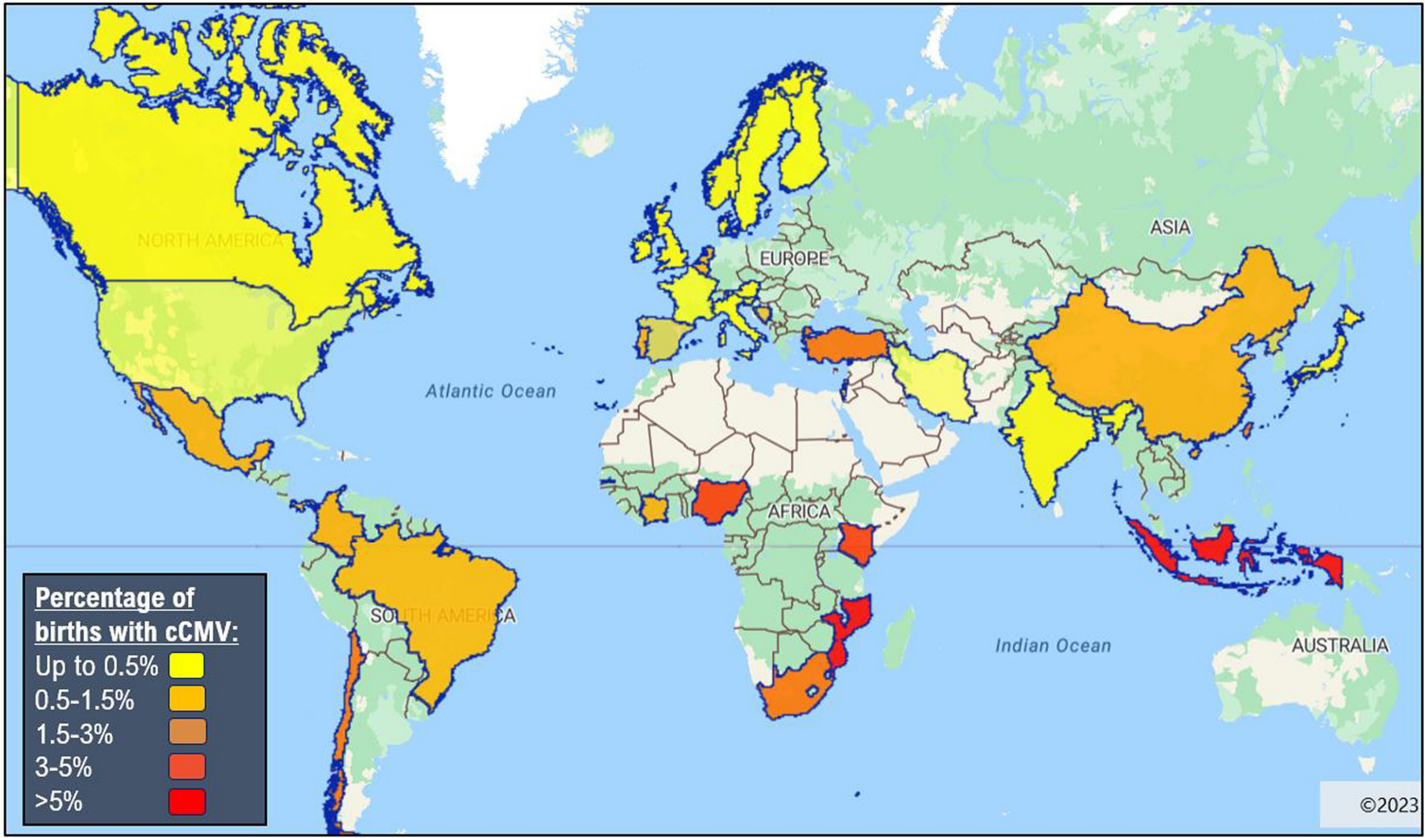
Global epidemiology of congenital cytomegalovirus (8, 17). Made with eSpatial.com^®^.

Global prevalence of cCMV is 0.67% (11). In Sub-Saharan Africa, the prevalence rates of cCMV ranges from 1.4% in the Ivory Coast (12) to 6% in Burkina Faso (13). The prevalence rates in individual countries vary considerably within this range, e.g., 5.4% in The Gambia (14), 3.8% in Nigeria (15), 3% in Uganda (12), 2.9–3.6% in Kenya (13, 14) and 2.5% in South Africa (16). It is notable that all are above the global cCMV prevalence rate of 0.67 (6), and even above the average for LMICs: 1.42% (0.97–2.08%) (8). However, there are numerous potential biases in these epidemiological studies and generalisability is limited since a relatively small number of Sub-Saharan countries have reported prevalence data (Figure 1). In addition, variance in the choice of screening tool and clinical sample used can impact results, as explained in more detail in the diagnostics section below. This is particularly true in retrospective studies using dried blood spots as the sensitivity of this assay can vary between 34 and 80% (18). There are also inherent biases in certain targeted screening studies, i.e., screening infants with hearing loss will not be representative of the population.

The prevalence of cCMV in preterm and very low birthweight infants is usually higher than in term infants with rates of 1.5–4.8% in HICs (19, 20), however, to the best of our knowledge, no data for cCMV in preterm births have been reported in Sub-Saharan Africa. The prevalence of cCMV is around 5% in infants exposed to HIV *in-utero* (20) and 6.5–11% (21–23) in infants with perinatally-acquired HIV. CMV-HIV co-infection is reported to lead to more rapid infant HIV disease progression (24, 25). Risk factors for cCMV transmission are recognised to include lower socio-economic status^11^, younger age of onset of sexual activity, maternal age less than 25 years, those caring for preschool children in the year before delivery, preterm labour, HIV co-infection and other sexually transmitted infections during pregnancy (26).

## Clinical presentation and long-term sequalae of cCMV

**Figure 2:**
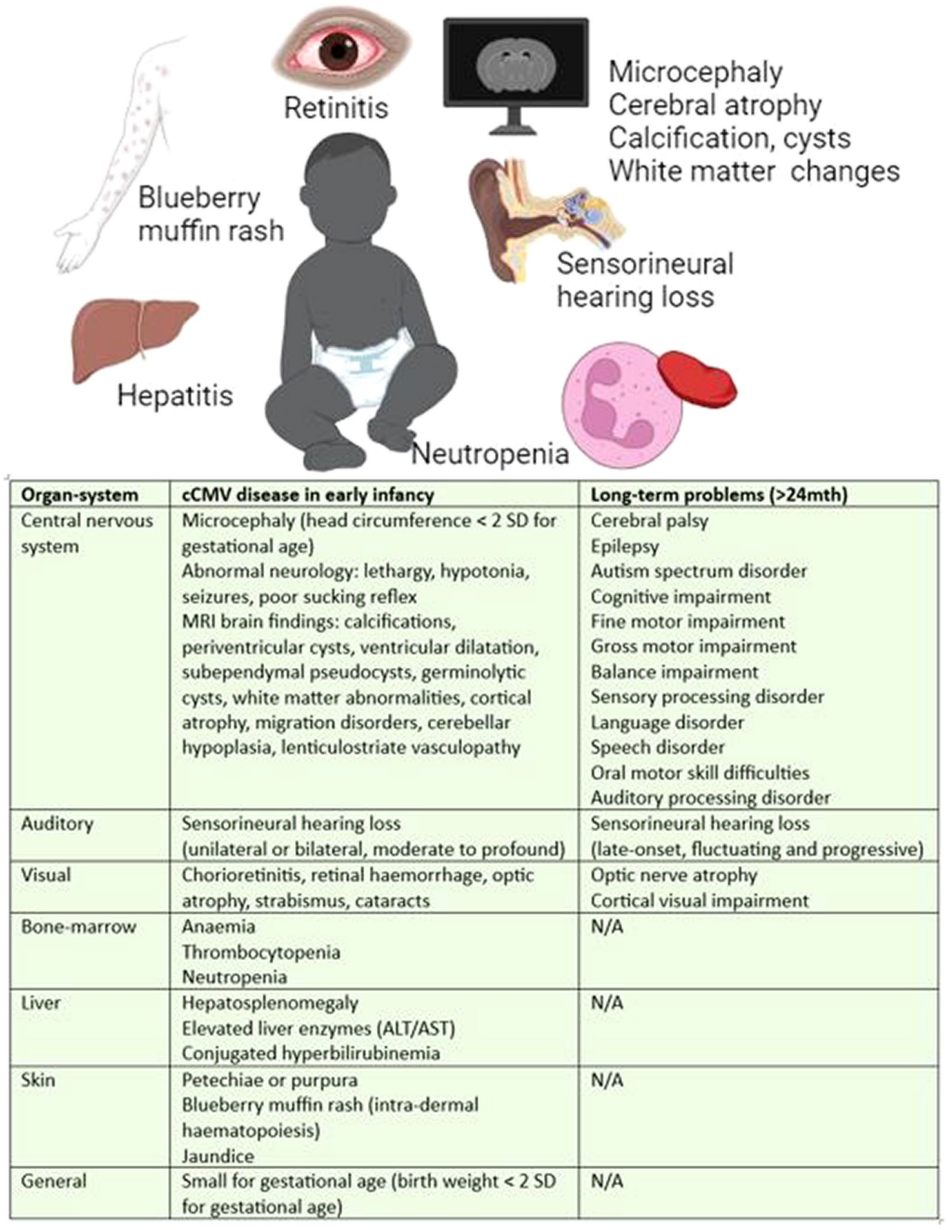
Manifestations of cCMV disease in infancy and long-term (27, 28). Long-term defined as >24 months of age.

Historically, cCMV infection has been classified as symptomatic or asymptomatic by the presence or absence of clinical findings on examination or basic blood tests, with symptomatic disease in 10–15% of infants. However, this paradigm is inconsistently applied in the literature, and becoming less meaningful as cases are increasingly being identified through targeted screening, lower thresholds for testing, and greater access to central nervous system (CNS) imaging in HICs. In infancy, cCMV causes growth restriction and a broad spectrum of multiorgan disease manifestations, including impairment of the CNS, vision, hearing, skin, liver and bone-marrow function. These features are determined by a combination of clinical assessment, laboratory, and radiological investigation. During infancy, features such as skin rashes, liver or bone-marrow impairment may resolve spontaneously or with treatment; and yet hearing loss, visual and neurological impairment can be longstanding and progressive, as described in Figure 2 (27, 28) cCMV is attributable to 10% of childhood cerebral palsy (29) and 25% of childhood deafness (30).

According to the historical classification, approximately 20% of asymptomatic and 50% of symptomatic neonates (28–32) with cCMV will develop long-term sequelae. This equates to around 350,000 infants in Sub-Saharan Africa each year, more than double the number of infants born with HIV^32^. Hearing loss is the most common long-term outcome with half of the symptomatic (29) and approximately 10% of asymptomatic neonates develop hearing loss (30). The presentation is unpredictable and can be unilateral or bilateral and stable, fluctuating or progressive (30). cCMV is the most common cause of hearing loss after genetic causes (33).

Various categories of cCMV disease have been proposed encompassing the spectrum of manifestations of cCMV. For example: (1) no CMV-related disease; (2) mild, isolated, or transient disease, e.g., jaundice, petechiae or intrauterine growth restriction; (3) moderate, with multiple minor findings; (4) CNS disease; (5) other severe, life-threatening, or severe non-CNS disease; or (6) isolated SNHL (27). One of the limitations of this categorisation is the wide range of manifestations that fall into the category of CNS disease, whereby microcephaly, calcification and white matter changes are given the same significance as polymicrogyria, lissencephaly, cortical, and cerebellar malformations. Neuroradiological scoring systems are being developed to better understand the significance of these findings (34, 35). However, the extent of developmental delay may not be fully appreciated until 4–6 years of age when social, communication and learning difficulties become evident (28). Furthermore, late-onset hearing loss can present up to 6 years of age (36).

## cCMV diagnostics

Early diagnosis is essential for initiation of treatment within the first month of life. This is currently the recommended timeframe supported by randomised data for symptomatic infants (37, 38). Symptomatic newborns are more likely to be tested for CMV, but up to 75% of these infants may have delayed diagnoses or be missed altogether since the signs and symptoms can be subtle and attributed to other causes (39). Without universal screening, many infants with mild or asymptomatic disease will be missed altogether. Some may be detected due to suspicion of CMV from maternal illness during pregnancy or testing for CMV in infants with SNHL.

Despite cCMV being the leading cause of neurodisability and hearing loss from a congenital infection, awareness within maternal and infant healthcare professionals remains as low as 23% (40–42), which will have a direct impact on rates of diagnosis and quality of care received. To date there are 17 publications reporting poor healthcare professional awareness of cCMV, however these are exclusively in HICs, and none report from Sub-Saharan Africa.

The paucity of data available from Sub-Saharan Africa is exacerbated due to difficulties in diagnosing cCMV, as diagnosis needs a polymerase chain reaction (PCR) from saliva or urine to be done within the first 3 weeks of life. These samples have a much higher viral load than blood (18) with urine having the highest sensitivity and specificity, but it is a less convenient sample to collect than saliva (43). Although saliva is easier, it may yield false-positive results if performed less than 1 h after a breast-feed. CMV PCR from blood is not helpful for diagnosis if negative because 25% of infants with cCMV do not have CMV viraemia (18). There is limited access to PCR diagnostics in most secondary healthcare facilities, particularly in rural settings. Samples can be sent to another institution to be processed but this may be costly and result in diagnostic delays. Serology is not helpful as CMV IgG will reflect maternal previous exposure to CMV and placental transfer of immunoglobulin, which is not protective for the infant. CMV IgM can reflect infant infection but has poor sensitivity (44), and so PCR remains the gold standard for diagnosis.

A positive PCR result collected from samples older than 3 weeks could indicate postnatal acquisition of infection. Postnatal CMV (pCMV) is commonly acquired through breast milk. In South Africa, 2% of term infants have acquired pCMV by 3 weeks and 21% by 3 months of age (45). In term infants pCMV is usually asymptomatic, but in preterm infants up to 15% of infants with pCMV have symptomatic disease which may include sepsis-like syndrome with respiratory distress, feed intolerance and potentially necrotising enterocolitis, jaundice, hepatitis, thrombocytopenia and neutropenia (45–47).

## Screening for cCMV

Serological screening of mothers in early pregnancy is practised in some settings of low CMV-seroprevalence as mothers with evidence of CMV-seroconversion could be offered treatment to prevent transmission to their unborn infant. Treatment with high-dose valaciclovir may be offered in early pregnancy since randomised controlled trial data suggests a 70% reduction of *in-utero* transmission of CMV (48). However, this is not yet widespread practice in HICs. Importantly for Sub-Saharan Africa, valaciclovir is unlikely to be helpful in countries with high CMV-seroprevalence because identification of non-primary infection is challenging. The use of CMV hyperimmune globulin may also be offered but incurs significant cost without convincing evidence of benefit (49).

Screening newborn infants for cCMV is not current practice in most countries, but pilot programmes have started recently in Canada and the Unites States using dried blood spot samples (11). Targeted screening of infants at higher risk of cCMV is practised in some HICs, e.g., screening of infants with growth restriction, born less than 30 weeks gestational age, or infants with failed newborn hearing screening (19, 20, 50, 51). However, these approaches to targeted screening are not practised in LMICs. Infants with perinatally-acquired HIV or exposed to HIV *in-utero* are at higher risk of cCMV, particularly in mothers newly diagnosed during pregnancy or with poor HIV virological control (21–23). These infants form another potential group for targeted screening along with any other immune deficiency. However, CMV diagnostic tests must be performed within 3 weeks of life to confidently differentiate between cCMV and pCMV, and the diagnosis of immune deficiency may not have been achieved in this timeframe. Differentiation between cCMV and pCMV could possibly be achieved clinically in term infants with typical features of cCMV, however immune-suppressed and very-low birth weight infants frequently acquire pCMV and determining postnatal from congenital infection can be difficult in these cases. Since the symptoms of cCMV can be non-specific and not always easily differentiated from other congenital infections, testing for cCMV should be considered as part of a clinician’s congenital infection screen (17).

## cCMV treatment

Treatment practice for cCMV is frequently guided by consensus statements from experts, however there are certain differences in expert opinion. The International Congenital Cytomegalovirus Recommendations Group was convened in 2015 to provide recommendations for prevention, diagnosis, and treatment. Treatment was only recommended for neonates with moderately to severely symptomatic cCMV disease (31). This is due to a lack of high-quality robust evidence for all of the above categories except CNS disease (37, 38). Subsequently, a European expert consensus statement published in 2017 (27), demonstrated majority opinion to treat isolated hearing deficit. Similarly, the Canadian Paediatric Society and the American Academy of Paediatrics have issued their respective practice statements (52, 53). These statements are representative of clinicians from the United States, Australia and Europe, and to date there have been no representation from Sub-Saharan Africa and no such consensus statement applicable to care deliverable within Sub-Saharan Africa. In addition, there have been no trials of treatment within an African setting.

Treatment for cCMV is based on two randomised controlled trials from the United States that demonstrated better audiological outcomes in symptomatic infants who were treated with 6 weeks of intravenous ganciclovir initiated within 28 days of life, compared to no treatment (37). The second trial compared from 6 months of oral valganciclovir versus 6 weeks (38) and demonstrated outcomes at 2 years were significantly better for hearing and highly significantly better for developmental, notably language and communication. However, despite treatment 23% of hearing remained abnormal or deteriorated. A subsequent observational study in Japan suggested treatment initiation for symptomatic infants between 1 and 2 months of age was non-inferior to treatment initiation at less than 1 month (54), but follow-up was limited to only 6 months.

Several studies have recorded around half of infants identified through screening have abnormal brain imaging including abnormal white matter and even polymicrogyria (34, 55). It is discussed whether infants with less severe disease might have a greater benefit from treatment than those more severely affected, however there are no randomised controlled trials to currently support treating infants with mild disease or isolated SNHL. The Toddler-Valgan randomised controlled trial examined starting treatment in children from 4 weeks to 4 years of age (40% were < 1 year of age) with CMV-associated hearing loss, 74% were considered symptomatic. The trial did not demonstrate improvement or stabilisation of hearing loss between 6 weeks of valganciclovir and placebo (56), but these were very small numbers, treatment was initiated up to 4 years of age, and outcome analysis was only at 6 months. In contrast, the Concert study, although not a randomised controlled trial, demonstrated improvement in hearing but not development at 20 months after 6 weeks treatment with valganciclovir in infants with isolated SNHL aged less than 12 weeks (57). Three other similar trials were terminated early due to poor recruitment or a safety signal (58–60).

Ganciclovir, or its oral derivative valganciclovir, are nucleoside inhibitors and are most commonly used for treatment of cCMV despite the fact that it is not licensed for infant use (31). Importantly, these treatments have side-effects of neutropenia and transaminitis and require at least monthly monitoring since adverse effects of neutropenia are observed in 21% of infants (38). However, neutropenia is also a feature of cCMV disease and it should be noted that during the trial comparing 6 weeks versus 6 months of valganciclovir, the rate of neutropenia did not differ significantly between the infants on treatment compared to those not on treatment beyond 6 weeks; thereby justifying its use despite the recognised high rate of side effects. Animal studies have inferred the possibility for oncogenesis and aspermatogenesis following use of ganciclovir, although the former has not been published and the latter was reversible (61, 62). Nevertheless, parents should be counselled regarding these potential side effects and long-term associations, particularly regarding the risk of neutropenia. If the infant develops fever, they should be seen by a medical practitioner without delay.

In LMICs access to oral valganciclovir is limited and expensive, especially in liquid formulation. The liquid formulation should be kept in the fridge, which may not be feasible in some homes. Alternatively, valganciclovir tablets can be crushed and resuspended in solution to be administered to infants (63, 64). Monitoring for neutropenia and transaminitis should be done 2 weeks after initiation of therapy and monthly thereafter. This requires parents to return to clinic and appropriately skilled healthcare professionals to take blood and follow-up accordingly with the results. Infants diagnosed with cCMV are therefore unlikely to receive the recommended 6 month duration of valganciclovir due to these limitations, and a pragmatic course of 6 weeks treatment are offered in some cases. In infants with both HIV and cCMV there is the additional challenge of managing multiple medications and it may be a case of prioritising anti-retroviral therapy in the first instance.

## cCMV follow-up

Due to the potential progressive nature of hearing loss in infants with cCMV, follow-up by audiology 3 monthly for 1 year, then 6 monthly until 3 years and finally yearly until 6 years is recommended. Cognitive, learning, speech, language, and social communication difficulties will not be evident until at least 1 year of age, therefore developmental assessment is recommended at 1 and 2 years, or more frequently if indicated. These assessments facilitate the timely involvement of appropriate allied health care professionals as required, e.g., physiotherapy, speech and language, occupational therapy, and educational support. However, infants are frequently not brought back to follow-up appointments due to travel, time or financial barriers, or lack of recognition of the presence of developmental or hearing issues that may be evolving in their child. Furthermore, access to allied healthcare support may be limited or unavailable, and is frequently not state-funded.

## Recommendations on the way forward for cCMV in sub-Saharan Africa

The burden of cCMV is under-recognised, under-resourced and under-treated in LMICs. There are numerous factors that contribute to poor outcomes of infants with cCMV. The core determinants are likely to be inconsistent awareness of relevant healthcare professionals, non-existence of maternal or neonatal screening, and limited access to diagnostics, medications, and allied healthcare support. Although there has been an increase in observational studies of cCMV in Sub-Saharan Africa, these remain low in numbers (6, 9). There is a stark absence of initiatives to enhance recognition, diagnosis and treatment (65), despite the burden of disease and disability from cCMV being equivalent to the number of infants born with HIV each year (66). The following recommendations have been made through review of relevant literature and informal discussions with paediatric infectious disease clinicians with experience managing cCMV in Sub-Saharan Africa. These recommendations represent a framework of strategies to begin to address the challenges described above.

### Recommendation 1: Develop a network of expertise

The first recommendation would be to develop a network of expertise within Sub-Saharan Africa which may be used as a platform for education, research and clinical support. This would require institutional endorsement, essential to facilitate access to appropriate diagnostics, therapeutics, and clinical care. The initial phases of establishing such a network would benefit from utilising existing neonatal care collaboratives or paediatric infectious diseases societies in the region such as the African Neonatal Association and the African Society for Paediatric Infectious Diseases (AfSPID). It would be valuable to work in collaboration with organisations such as cCMVNET, an international network of paediatricians, neonatologists and scientists conducting clinically orientated research in cCMV, and supporting clinical education. While utilising the strengths of existing networks, the importance of establishing a Sub-Saharan network lies in developing strategies that rightly address regionally specific challenges.

### Recommendation 2: A consensus statement from experts within sub-Saharan Africa

The first role of the regional network would be to develop a consensus statement on the most appropriate approach to investigate, diagnose, and manage cCMV within current contextual limitations, as described above. The consensus statement should establish a pragmatic approach to diagnosis and management that can be implemented in most LMICs, and should include advice to be given to pregnant women to reduce CMV acquisition in pregnancy. Such a consensus statement could be achieved using a Delphi Method.

### Recommendation 3: Raise public and healthcare professional awareness

From this standpoint raising public and healthcare professional awareness is critical, particularly within the key departments of obstetrics, midwifery, neonatology, paediatrics, audiology, ophthalmology, and developmental paediatrics. The network could facilitate communication between expert clinicians on the optimal management of difficult cases and could integrate with the existing cCMVNET which holds monthly online seminars and case discussions. The importance of raising public awareness is in attempt to prevent acquisition of CMV during pregnancy by encouraging parents to increase hand hygiene, give forehead kisses, and not to share cutlery, straws or pacifiers that may have been in contact with saliva. Semi-structured interviews of pregnant women demonstrated a strong desire to be informed about CMV as part of routine antenatal care, and that expressing behaviour changes as risk reduction rather than prevention, made the behaviours feel realistic and potentially achievable (67).

### Recommendation 4: Targeted screening for high-risk infants

With consensus on clinical approach to investigation and management, and improved clinician awareness, it may be possible to initiate targeted screening programmes. Suggested groups for targeted screening should be infants with failed hearing screening, premature or very low birth-weight infants, or those with diagnosis of perinatally-acquired HIV or other immune deficiency. Newborn hearing screening provides an important opportunity to identify asymptomatic infants with cCMV, who would not have been identified by routine clinical examination. Targeted testing of infants for cCMV may be offered for infants who have no clear responses on the newborn hearing screen (49, 50), which is an approach that ensures a diagnosis is made within a suitable timeframe to enable antiviral treatment to be initiated. Routine targeted testing for CMV in Utah and Connecticut in the United States, has been shown to be cost-effective (68).

### Recommendation 5: Research strategies

Published research on cCMV in Sub-Saharan Africa are largely observational, and there is limited data on screening, diagnostics, or interventional approaches in this setting. The final recommendation is to develop research capacity across the region utilising the clinical network as per recommendation 1 to develop an infrastructure from which high-quality, contextually appropriate clinical studies can be conducted. Major areas of research should include developing diagnostics, treatment algorithms and clinical trials that address the need for pragmatic treatment strategies in this context. Inexpensive point of care diagnostics, which are also clinically acceptable to be used as a screening tool in targeted screening programmes would revolutionise the diagnostic process. Data collected from across the network could be used to formulate algorithms from clinical information typically available to predict those that will have long-term and late-onset sequalae, and therefore rationalise treatment to those who would most likely benefit. Finally clinical trials should be conducted to explore potential effectiveness of reduced treatment duration stratified by risk, a pragmatic approach to treatment duration in consideration of the challenges of access to medication, monitoring and limiting risk of side effects.

For the last two decades, it has been a high priority to develop a vaccine against CMV, and there are numerous candidate CMV vaccines in development targeted to prevent congenital and post-transplant infection. There is preliminary data from Phase II trials that vaccination might prevent acquisition of CMV in seronegative women exposed to CMV, and several candidate vaccines aimed at the goal of preventing congenital CMV disease are moving towards Phase III trials (69). In due course it may be appropriate to consider implementing trials to demonstrate the potential benefit of CMV vaccines in the prevention of cCMV in a high seroprevalence setting.

With growing recognition of the enormous burden of cCMV within Sub-Saharan Africa, it is most certainly time for a call to action to approach strategies for prevention in pregnancy, promote education within healthcare professionals, address issues of diagnostics, implement screening, and improve treatment strategies (Table 1). Our primary recommendation is to establish a regional network of expertise to establish a consensus on investigation and management within Sub-Saharan Africa which can be a platform for education, research, and clinical support to thrive, and ultimately improve the health and well-being of our future infants.

**Table 1:** Table of recommendations for addressing issues of cCMV infection in sub-Saharan Africa.

Recommendations
Develop a network of expertise within sub-Saharan Africa which may be used as a platform for education, research and clinical excellence. Create a consensus guideline of cCMV diagnosis and management within contextual limitations. Raise clinician and public awareness. Targeted screening programmes: infants with hearing loss, HIV-exposure and infection, prematurity and low-birth weight. Research focus: Inexpensive point of care diagnostics. Formulate algorithms using clinical features to anticipate which infants will have long-term sequalae and most likely benefit from treatment. Trials to demonstrate effectiveness of treatment stratification based on risk.

## Author contributions

HP: Writing – original draft. SB: Writing – original draft.
